# Response to Zona, E.E.; Israel, J.S. Toward Individualized Management: A Commentary on Perioperative Systemic Therapy Guidelines in Breast Cancer Surgery and Reconstruction. Comment on “Galuia et al. Perioperative Drug Management of Systemic Therapies in Breast Cancer: A Literature Review and Treatment Recommendations. *Curr. Oncol.* 2025, *32*, 154”

**DOI:** 10.3390/curroncol33080463

**Published:** 2026-08-03

**Authors:** Mariem Galuia, Julia Fedorova, Eleftherios Mamounas, Sabrina Pavri, Sarfraz Ahmad, Wassim Mchayleh

**Affiliations:** 1 Department of Internal Medicine, AdventHealth Hospital, Orlando, FL 32804, USA; 2 College of Medicine, University of Central Florida, Orlando, FL 32827, USA; 3 Division of Breast Surgery, Department of Surgery, AdventHealth Hospital, Orlando, FL 32804, USA; terry.mamounas.md@adventhealth.com; 4 Division of Plastic Surgery, Department of Surgery, AdventHealth Hospital, Orlando, FL 32804, USA; sabrina.pavri.md@adventhealth.com; 5 Gynecologic Oncology Program, AdventHealth Cancer Institute, Orlando, FL 32804, USA; sarfraz.ahmad@adventhealth.com; 6 Department of Medical Oncology, AdventHealth Hospital, Altamonte Springs, FL 32701, USA; wassim.mchayleh.md@adventhealth.com

We thank Dr. Zona and Dr. Israel for their thoughtful commentary on our article and for their important contributions to the evolving area of perioperative management in breast cancer [1].

We also read with great interest their recent publication in Plastic and Reconstructive Surgery, which provides a systematic review and multidisciplinary expert consensus on the perioperative management of anticancer agents in the context of breast reconstruction. Their work offers a clinically relevant recommendation panel that integrates available evidence with multidisciplinary expertise.

Indeed, both their work and ours aims to address an important gap in the absence of high-quality data. However, our approaches appear to differ in a few points. From a methodology standpoint, we integrated preclinical and early clinical data, including pharmacokinetic principles such as drug half-lives and clearance, with field experts’ experience, to propose standardized and reproducible recommendations for perioperative drug holds [2]. As discussed in our review, these recommendations were developed in the setting of limited prospective perioperative trials and therefore should be interpreted in the context of patient-centered, individualized decision-making [2]. In contrast, Zona et al. employed a systematic review combined with multidisciplinary consensus, and included clinical parameters such as hematologic adverse effects into their recommendations. We believe both approaches are complementary rather than conflicting. To illustrate the matter, we can cite a few examples that consolidate this statement:

First, for tamoxifen, our recommendations primarily informed by its prolonged half-life and pharmacologic persistence while keeping in sight the critically important thromboembolic risk. Zona et al., while also acknowledging these pharmacologic properties, recommend holding tamoxifen for 2 weeks prior to major reconstruction, similarly integrating both pharmacokinetics and clinical concerns such as thromboembolic risk and wound healing complications. 

Second, for PARP inhibitors such as Olaparib, their recommendations incorporate both timing (such as holding therapy 48 h preoperatively) and laboratory monitoring, reflecting awareness of hematologic toxicity, neutropenia, and infection risk, all of which may influence surgical recovery and wound healing [2,3]. These further underscore the importance of integrating pharmacologic and clinical considerations.

Third and most importantly, for HER2-targeted drugs such as trastuzumab and pertuzumab, we both agree that routine perioperative discontinuation is not recommended. This point reflects reassuring safety data and highlights areas of agreement between our recommendations.

Zona et al. for introduced an important point; the distinction between major and minor procedures in terms of treatment management, recommending more conservative management for complex operations such as autologous reconstruction, while allowing more flexibility for minor procedures. For example, temporary holding systemic therapy appears to be more plausible and more strongly considered for extensive flap reconstruction requiring prolonged operative time and tissue healing. On the other hand, minor procedures, such as implant exchange, fat grafting, and limited surgical revisions, may permit a shorter interruption duration depending on the patient’s clinical profile and surgical risk. This stratification further emphasizes our individualized perioperative planning, and we thank the authors for commenting on the matter.

An additional important point to consider is the difference in recommendations between immediate and delayed reconstruction. While delayed reconstruction generally allows greater scheduling flexibility and patient clinical recovery prior to surgery, immediate reconstruction may require careful decisions balancing treatment continuity and efficiency with perioperative risks related to wound healing.

Differences in drug hold durations between our articles (which were generally shorter in Zona et al.’s approach), likely reflect the above-described different methodological frameworks. While we prioritized consistency and reproducibility based on pharmacologic properties in light of experts’ multidisciplinary experience [2], their recommendations appear more individualized care informed by clinical context. Nevertheless, both approaches converge on the shared ultimate goal of enhancing oncologic safety while obtaining optimal surgical outcomes.

In summary, there is no universally accepted standard for the perioperative management of systemic therapies in breast cancer, largely due to the limited availability of prospective surgical data for many recently approved oncologic drugs. Continued interdisciplinary collaboration and prospective research are needed. We thank the authors for advancing this important dialog and for their valuable contribution to the field.

## Data Availability

No new data were created or analyzed in this study. Data sharing is not applicable to this article.
